# The impact of robotic surgery on reducing radiation exposure in orthopedic trauma: a meta-analysis

**DOI:** 10.1007/s11701-025-02454-7

**Published:** 2025-06-23

**Authors:** Marzieh S. Saeedi-Hosseiny, Hanna G. Rothenberg, Aziza L. Jadallah, Umar Khaja, Allen Karimov, Mohammad H. Abedin-Nasab

**Affiliations:** 1 Biomedical Engineering Department, Rowan University, Glassboro, NJ USA; 2 Virtua Health College of Medicine and Life Sciences, Rowan University, Stratford, NJ USA

**Keywords:** Radiation time, Robotic surgery, Minimally invasive surgery, Orthopedic trauma, Fluoroscopy time

## Abstract

Radiation time is a critical metric influencing safety for both healthcare providers and patients during minimally invasive orthopedic trauma surgeries. This meta-analysis aimed to compare total radiation time between robotic guidance and manual fluoroscopy, while also compiling global statistics on operative radiation exposure, associated health risks, and compliance with protective measures. Relevant comparative studies were identified through comprehensive searches in PubMed, Scopus, Web of Science, Medline, ClinicalKey, and Embase. Ten studies, encompassing 675 patients, met the inclusion criteria. Data on treatment groups, procedure success rates, robotic systems used, and other pertinent variables were systematically extracted and reviewed following the Preferred Reporting Items for Systematic Reviews and Meta-Analyses (PRISMA) guidelines. A random-effects model in SPSS was applied to analyze total radiation time. Results revealed a significantly shorter radiation time with robotic guidance, supported by a robust effect size, fragility index, and fragility quotient. These findings suggest that robotic systems may offer significant safety advantages. Future research should explore the broader implementation of robotic guidance and its implications for patient and provider safety across various surgical fields.

## Introduction

Minimally invasive orthopedic trauma surgery relies heavily on intraoperative fluoroscopy, which presents a significant occupational hazard due to prolonged radiation exposure [1–3]. Among orthopedic subspecialists, trauma surgeons are exposed to the highest cumulative doses, contributing to elevated risks of cancer and other radiation-related complications [1, 2]. Despite the availability of protective gear, compliance is often inconsistent, and even full use of standard shielding does not adequately protect vulnerable anatomical areas such as the head, neck, and breasts [3–5]. These concerns have prompted increased interest in developing safer, more efficient imaging and guidance technologies.

Robotic surgical systems have emerged as a promising alternative, offering enhanced precision and the potential to reduce reliance on repeated fluoroscopic imaging. By integrating preoperative imaging, real-time navigation, and autonomous trajectory planning, robotic platforms can streamline intraoperative workflows and reduce procedural fluoroscopy time [6, 7]. In orthopedic trauma procedures, which often involve narrow anatomical corridors and complex bony trajectories, robotic assistance may enhance targeting accuracy while simultaneously decreasing radiation exposure [8, 9]. Accuracy not only enhances outcomes, but also reduces risk. A 2022 randomized controlled trial reported that robot-assisted screw placement in lumbar surgery resulted in significantly less intraoperative blood loss compared to fluoroscopy-guided placement [10]. Further, a 2017 randomized controlled trial demonstrated a shorter average hospital stay of 6.8 days following robot-guided spinal fusion, compared to 9.4 days for fluoroscopy-guided procedures, representing a reduction of approximately 28% in hospital stay [11].

Several studies have shown that robotic guidance significantly reduces fluoroscopy time in procedures such as pedicle screw placement, sacroiliac fixation, and cannulated screw insertion [8, 9, 12]. However, some comparative studies note longer setup times, with total operative durations similar to those of manual fluoroscopy-guided techniques [13–15]. These findings suggest that workflow demands may offset some efficiency gains. Still, robotic systems offer notable safety advantages: the MIS ReFRESH trial found a 5.8-fold reduction in surgical complications and an 11-fold reduction in revision surgeries compared to traditional methods [16]. While conventional techniques may retain advantages in speed in some cases, robotic surgery presents a compelling case for integration, especially in high-risk or complex scenarios.

However, while robotic systems show promise in reducing radiation exposure, most existing studies quantify this effect only in terms of total fluoroscopy time. Most available studies report only total fluoroscopy time, used as a proxy for radiation exposure, rather than measuring absorbed dose or dose–area product (DAP), which are more accurate indicators of biological risk [3, 17]. Additional variables such as OR setup, imaging settings, patient body habitus, and distance from the radiation source also influence dose exposure, complicating direct comparisons [3, 17].

To address these limitations and synthesize the available evidence, this systematic review and meta-analysis aims to evaluate whether robot-assisted orthopedic trauma procedures are associated with reduced fluoroscopy time compared to conventional manual fluoroscopic techniques. By aggregating data from diverse study designs and clinical settings, this work seeks to clarify the safety advantages of robotic guidance in minimizing radiation exposure and to inform future decisions on technology adoption in orthopedic trauma care.

### Radiation safety in the operating room

Orthopedic trauma surgery often requires extensive use of fluoroscopy, placing surgical teams at considerable occupational risk due to cumulative radiation exposure. Among all orthopedic subspecialties, trauma and deformity surgeons experience the highest doses of radiation, which correlates with increased long-term cancer risk [1, 2]. Female surgeons are particularly vulnerable, with data showing a significantly elevated incidence of breast cancer compared to their peers in other surgical disciplines (Table 1) [18].

**Table 1:** Rates of overall cancer and breast cancer in surgical subspecialties, ranked by radiation exposure: greatest to least [18]

Surgical specialty	Female surgeons (*n* = 1185)	Expected cancer rate*	Actual cancer rate	Expected breast cancer rate*	Actual breast cancer rate	*p* value
Orthopedic	505	2.6%	**4.8%**	1.1%	**3.2%**	< 0.001
Urologic	328	2.7%	2.1%	2%	1%	> 0.3
Plastic	352	4.5%	4.0%	1.1%	1.4%	> 0.3

^1^By demographic cohort: age, race, and gender

Concerns are especially acute for pregnant orthopedic surgeons. Even with standard protective gear, fetal exposure can exceed recommended safety thresholds set by the International Commission on Radiological Protection (ICRP), which advises limiting total fetal exposure to no more than 1 mSv throughout pregnancy [1–3]. This threshold can be surpassed after just a few high-dose procedures [3]. Despite these risks, compliance with protective protocols remains inconsistent across operating rooms, and existing equipment does not fully shield critical organs such as the thyroid, breasts, and colon [4, 5].

These concerns are supported by procedural exposure data: orthopedic surgeons may absorb up to 1084 mGy annually, over 50 times the recommended limit, while patients themselves receive far greater doses per procedure (Table 2) [2, 4, 17]

**Table 2:** Radiation exposure and protective gear compliance in orthopedic surgeons and their patients [2, 4, 17]

Radiation exposure	Dose per procedure (mGy)	Annual procedures	Annual dose (mGy)	Recommended limit (mGy)	Gear compliance
Patient	178.3	1–2	356.6	1	100%
Surgeon	0.16–6	352.8	1084.2	20	45%
Surgeon’s child in utero	0.07	264.6	18.5	1 (ICRP, NCRP)	Not reported

^1^Each millisievert (mSv) of radiation received corresponds to approximately one milligray (mGy) of absorbed dose

^2^Radiation doses reported refer to exposure above the natural background level of approximately 3 mSv per year

Reducing radiation exposure is, therefore, not only a technological challenge, but a clinical imperative. While improving protective compliance is important, the most direct and scalable mitigation strategy is to reduce total fluoroscopy time during procedures. The literature consistently shows that radiation exposure declines exponentially with distance from the beam source and that procedure time is the strongest predictor of cumulative exposure [3].

Fluoroscopy time is directly correlated with patient radiation exposure, as quantified by metrics such as the dose–area product (DAP) [19], kerma–area product (KAP) [20], and entrance-surface dose (ESD) [21].

## Materials and methods

A systematic review and meta-analysis were conducted following the PRISMA 2020 guidelines [19].

### Inclusion criteria

Primary clinical data were collected on the MIS for orthopedic trauma. Studies comparing radiation time under robotic or manual fluoroscopic guidance under controlled or matched conditions were included. Data collection was limited to studies from 2000 onward to reflect contemporary surgical robotics approved for human use. This dataset included global studies across various nations and languages. Appropriate study types included randomized controlled trials (RCTs), cohort studies, prospective cohorts, retrospective reviews, and case-controlled studies.

### Exclusion criteria

Studies involving animals, cadavers, and animal cadaver models were excluded. Additionally, studies on cardiac or gastrointestinal procedures, or orthopedic procedures that were not minimally invasive, not comparative, or did not have full text available were excluded. Background articles were also removed. Studies that compared robotic and manual fluoroscopic trauma MIS, but did not report radiation time were excluded.

### Information sources and search strategy

Six major medical databases were searched: PubMed, Scopus, Web of Science, Medline, ClinicalKey, and Embase. Search terms included:

- [(Robotic surgery OR Robotics OR Robotic-assisted surgery OR Robotic enhanced surgery OR Robotic surgical procedures)
- (Fluoroscopy OR Fluoroscopic OR Fluoroscopy guided OR Fluoroscopic guidance)
- (Orthopedic surgery OR Orthopedics OR Orthopedic procedures OR Orthopedic surgeries OR Orthopedic surgical procedure OR Orthopedic surgical procedures)
- (Radiation time OR Fluoroscopy time)].

This general structure was applied uniformly across all databases to maintain consistency. Rayyan.ai software was used to identify potential duplicates, which were then independently reviewed by each author. Duplicates were removed, and the remaining articles were manually screened for eligibility and uniqueness.

### Study selection

**Fig. 1:**
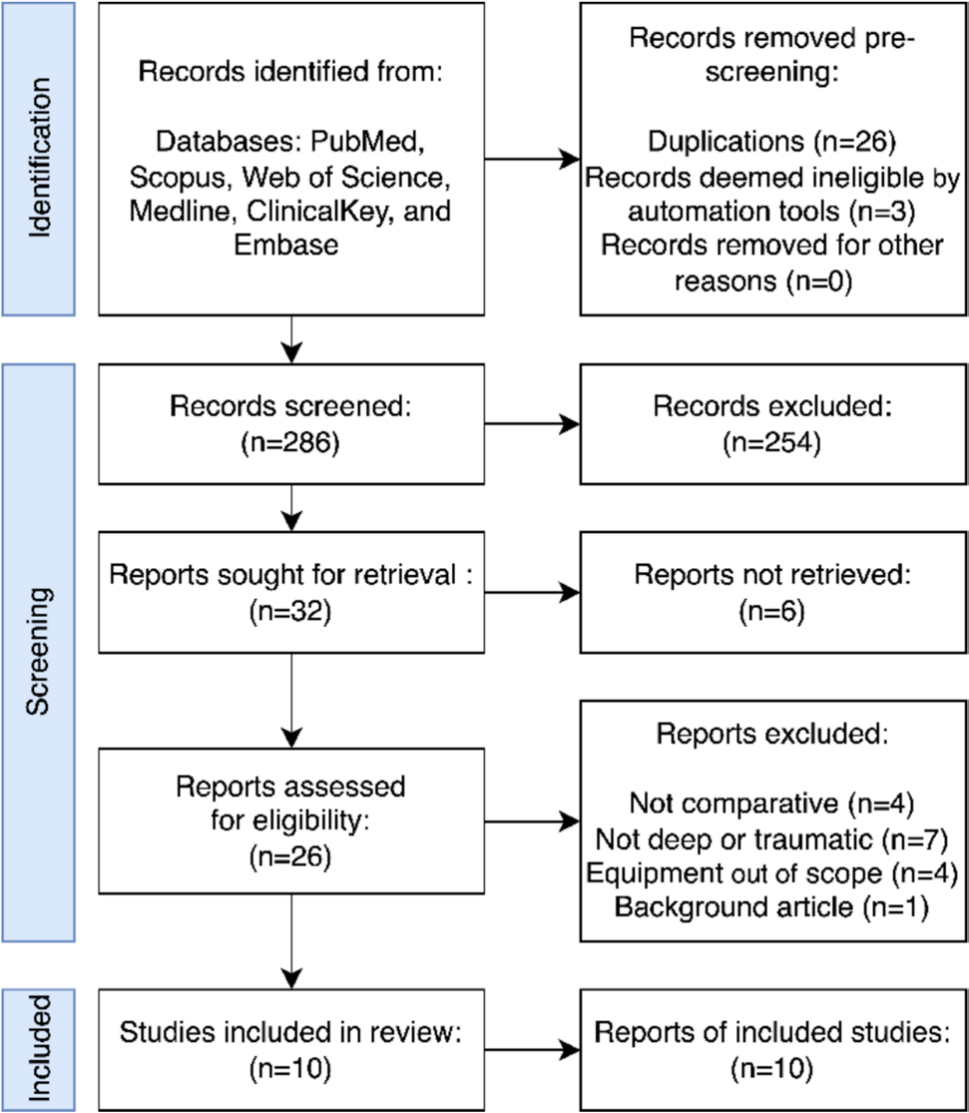
Flowchart depicting the systematic process of study identification, including applying inclusion and exclusion criteria in study selection

After confirming that all articles were unique, the abstracts were analyzed for inclusion. The team then appraised the full text of qualifying studies for detailed evaluation. Studies comparing radiation time were selected, and their frameworks were compared for prospective bias (Fig. 1).

### Data collection

Radiation time data for robot-guided and manual fluoroscopic MIS in orthopedic trauma were extracted. Table 3 presents the study-specific mean radiation times, standard deviations, and patient sample sizes [7–9, 12, 13, 20–24]. A random-effects meta-analysis was performed using IBM SPSS Statistics 29.0 to assess differences in radiation time. The qualitative analysis examined the algorithms and robotic systems used, statistical significance, data collection methods, and the generalizability of findings to various orthopedic patient subtypes. These insights provided the foundation for a comprehensive critique of each study’s design.

**Table 3:** Mean radiation time and standard deviation for robot-guided and manual fluoroscopic procedures [7–9, 12, 13, 20–24]

Study	Mean-R (s)	SD-R (s)	Mean-M (s)	SD-M (s)	No. of patients (R)	No. of patients (M)
[7]	16.95	7.35	42.42	7.35	30	30
[8]	29.40	2.40	57.00	9.60	56	56
[21]	334.80	73.20	435.00	50.40	37	44
[22]	352.80	77.40	663.00	178.80	56	35
[9]	75.00	5.40	81.60	6.00	65	61
[23]	435.00	191.40	958.20	309.00	38	25
[13]	58.80	23.40	56.40	28.80	26	24
[24]	24.00	25.80	211.20	111.00	10	17
[12]	348.00	261.00	672.00	252.00	10	10
[20]	360.00	816.00	2160.00	816.00	23	22

*Note*: some standard deviations were only given as an average of both treatments’ standard deviations, so this average was used for both

^1^R = robot guided, M = manual fluoroscopic

^2^Mean and standard deviation (SD) are measured in seconds (s)

^3^No. of patients = number of patients per procedure type

### Assessing evidence for certainty and risk of bias

The selected studies were evaluated for certainty of evidence using the Grading of Recommendations Assessment, Development, and Evaluation (GRADE) criteria. Two team members independently applied the GRADE framework. Bias was assessed using the study design: controlled case studies were evaluated using the Newcastle–Ottawa Scale, and randomized controlled trials were evaluated using Cochrane’s risk of bias (RoB) for randomized trials (RoB2). Consensus on the results is presented in Table 4 [19].

**Table 4:** GRADE assessment of certainty of evidence and risk of bias in selected studies [7–9, 12, 13, 20–24]

Study	Study design	No. of patients	System used	Limitations	RoB	Imprecision	Initial grade	Final grade	Importance
[7]	Randomized trial	60: 30 (R) 30 (M)	TiRobot (Beijing, China)	Not serious, due to demographically controlled groups	Low	Low: 99% CI	I	I	High
[8]	Retrospective cohort	112: 56 (R) 56 (M)	TiRobot	Somewhat serious, due to excluding patients 65 + years, severe osteoporosis, or bone disorders difficult to treat	Low	Low: 99% CI	III	III	Moderate
[21]	Retrospective cohort	81: 37 (R) 44 (M)	TiRobot	Somewhat serious, due to excluding patients < 55 years, or above the lowest 5% of intact bone mineral density	Low	Low: 95% CI	III	III	Moderate
[22]	Prospective cohort	91: 56 (R) 35 (M)	TiRobot	Not serious, due to demographically controlled groups	Low	Low: 95% CI	II	II	High
[9]	Retrospective cohort	126: 65 (R) 61 (M)	TiRobot	Not serious, due to demographically controlled groups	Low	Low: 95% CI	III	III	Moderate
[23]	Retrospective cohort	63: 38 (R) 25 (M)	TiRobot	Not serious, due to demographically controlled groups	Low	Low: 99% CI	III	III	Moderate
[13]	Randomized trial	50: 26 (R) 24 (M)	ADAPT (Freiburg, Germany)	Not serious, due to demographically controlled groups	Low	High: 66% CI radiation time	I	II	Low: imprecise CI for radiation time
[24]	Case–control study	27: 10 (R) 17 (M)	Brainlab Airo (Munich, Germany)	Not serious, due to controlled case cohorts	Low	Low: 99% CI	IV	IV	Low
[12]	Prospective cohort	20: 10 (R) 10 (M)	Pedi-Guard	Not serious, due to demographically controlled groups	Low	Low: 99% CI	II	II	High
[20]	Prospective cohort	45: 23 (R) 22 (M)	TiRobot	Not serious, due to demographically controlled groups	Low	Low: 99% CI	II	II	High

^1^Robot-guided surgery patients (R), manual fluoroscopy-guided surgery patients (M)

^2^Inconsistency and indirectness were not serious for all studies, except Wang 2023 [8], which had serious indirectness

### Data analysis

**Fig. 2:**
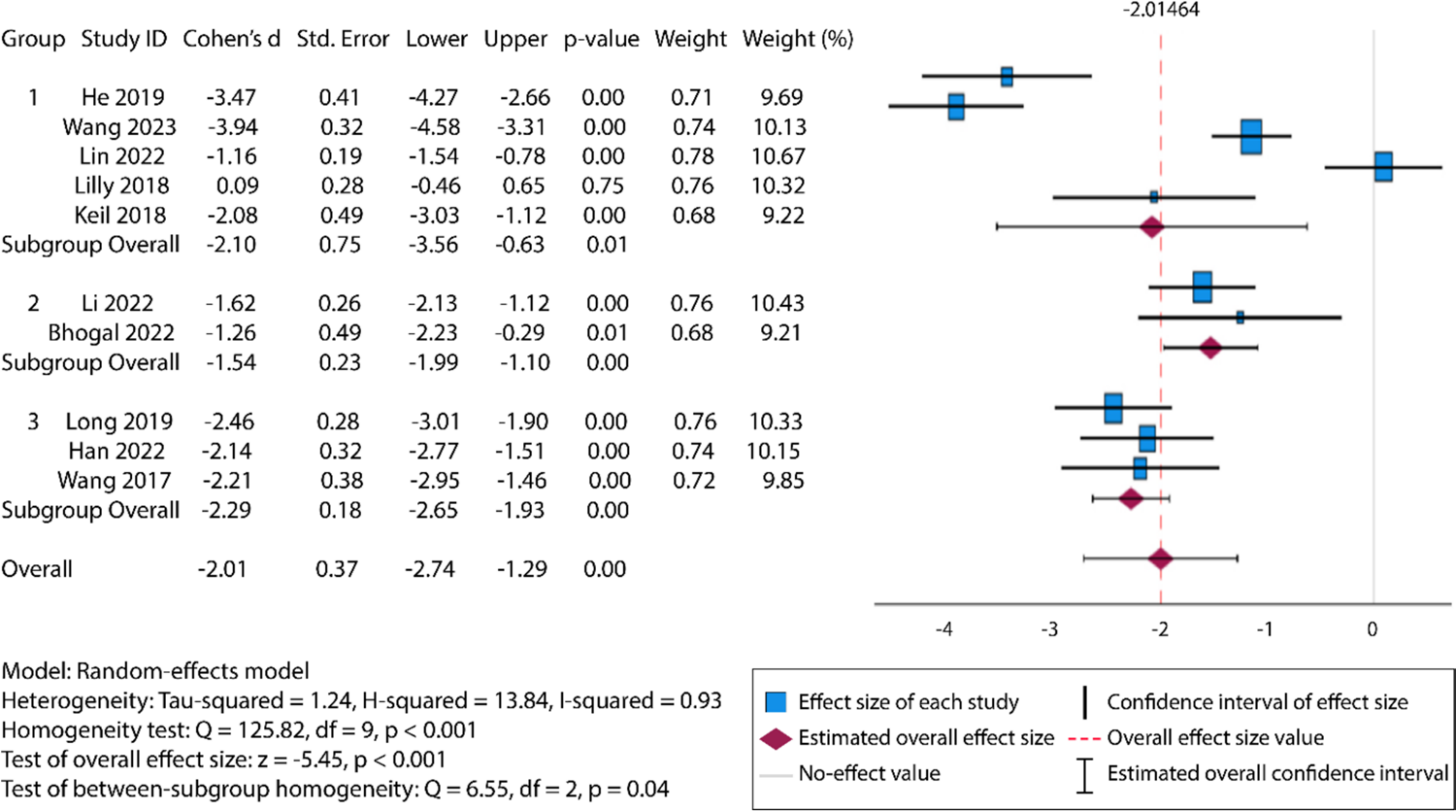
Forest plot depicting the random-effects meta-analysis and standardized effect size (Cohen’s *d*) for each included study, grouped into three predefined subgroups. A minimum significant effect size is defined as 0.2, while a large effect size is defined as ≥ 0.8 [26]. The overall pooled effect size of − 2.01 indicates a highly significant reduction in radiation time for robot-guided procedures compared to conventional techniques. The lower limit represents the maximum expected reduction in radiation time due to robotic guidance, while the upper limit reflects the minimum expected reduction or possible increase in some cases. The analysis revealed substantial heterogeneity (*I*^2^ = 93%, *p* < 0.001) and significant subgroup differences (*Q* = 6.55, *df* = 2, *p* = 0.04)

Statistical analysis was conducted using SPSS, version 29.0 (Chicago, USA). A random-effects model was applied to estimate the difference in total radiation time between robot-guided and fluoroscopy-guided MIS for orthopedic trauma. The effect size was found to be significant, as shown in Fig. 2. The effect size’s standard deviation was incorporated into the Chi-squared ratio, indicating overall variance between studies. To further validate statistical significance, the fragility index [25] was calculated using a clinical formula, utilizing the online Fragility Index Calculator (https://clincalc.com/Stats/FragilityIndex.aspx).

The ten studies included were subdivided by the type of procedure into three subgroups. Group 1 addressed fracture of the femur or its socket, the acetabulum. Group 2 explored multisegment vertebral fusion. Group 3 examined posterior pelvic ring fracture and its fixation via sacroiliac screw placement. Each subgroup had a large (> 0.8) effect size in favor of fluoroscopy time under robot guidance. At *p* < 0.01 per subgroup, Group 1 had a Cohen’s *d* of − 2.10 [− 3.56, − 0.63], Group 2 had a Cohen’s *d* of − 1.54 [− 1.99, − 1.10], and Group 3 had a Cohen’s *d* of − 2.29 [− 2.65, − 1.93]. Heterogeneity within each group was generally minimal. Groups 2 and 3 each had *I*^2^ = 0%, while Group 1 had a high heterogeneity at *I*^2^ = 96.5%, possibly due to the noted variation in femoral anatomy and its exploration.

**Fig. 3:**
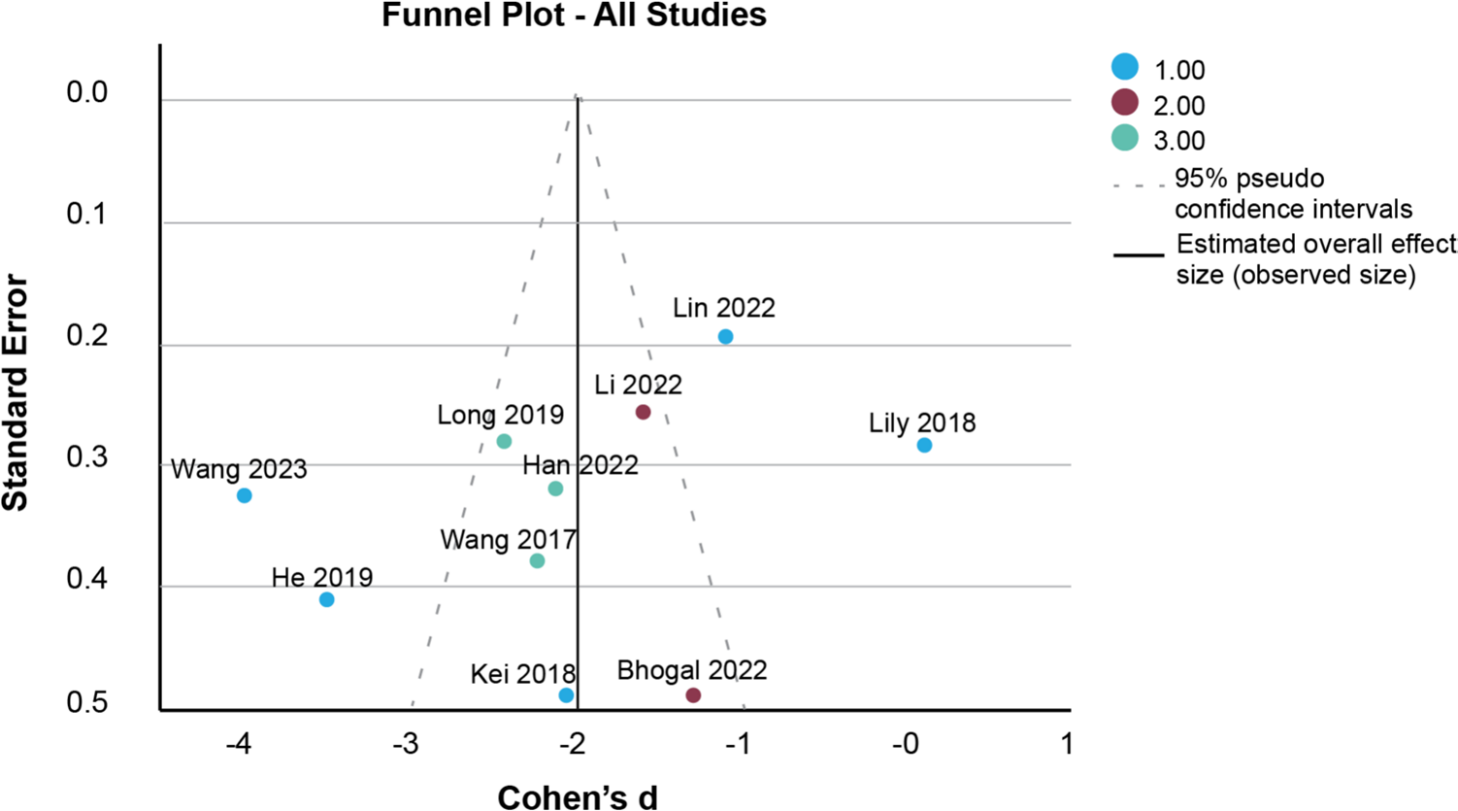
Funnel plot of all included studies. The vertical line indicates the pooled effect size; dashed lines represent 95% pseudo confidence limits. Symmetry suggests a low risk of publication bias

Figure 3 plots the effect distribution for publication bias, which summarizes bias due to heterogeneity or small sample bias. In small samples, bias, an effect size made disproportionately large by a small sample. The 95% confidence interval of effect size is distributed across − 3 to − 1 Cohen’s *d*. Ultimately, no bias due to heterogeneity or small sample size was detected.

**Fig. 4:**
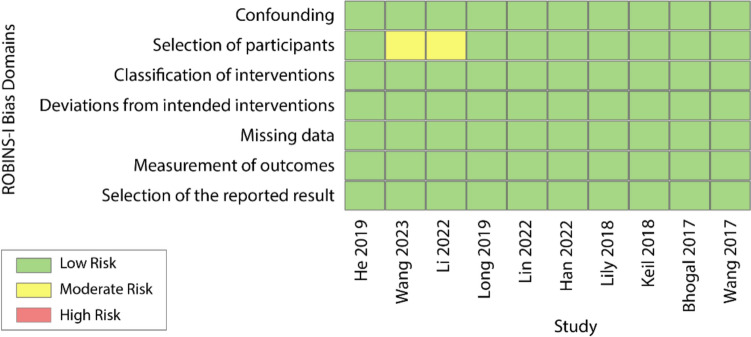
Risk of bias summary using ROBINS-I domains. This traffic-light summary visualizes the risk of bias across ten included studies using the ROBINS-I framework. Each row represents a study, and each column represents a bias domain. Colors reflect assessed risk levels: green = low risk, yellow = moderate risk, and red = high risk. Most studies demonstrated low risk across all domains, with moderate risk identified only in participant selection for two retrospective cohort studies

**Fig. 5:**
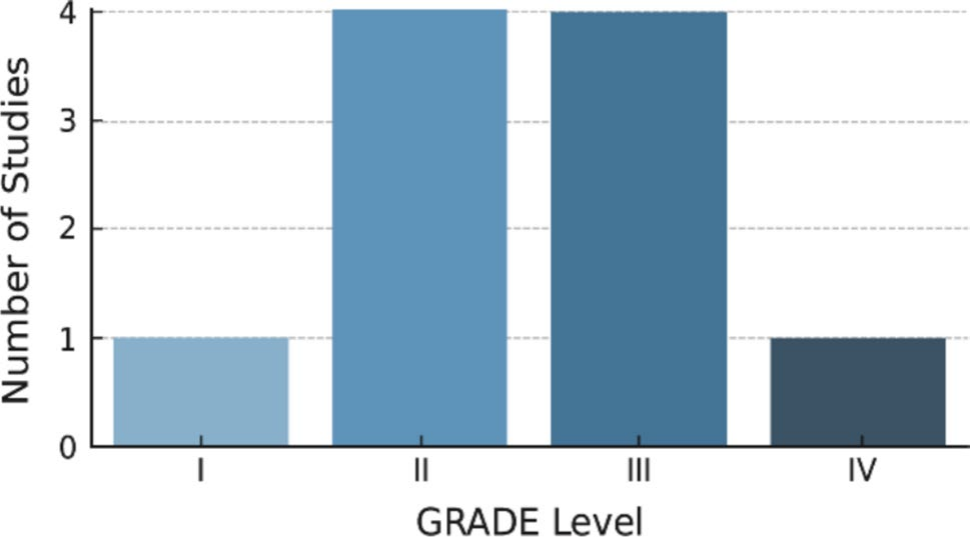
Final GRADE certainty of evidence across included studies. Most studies were classified as Level II (*n* = 4) or Level III (*n* = 4), indicating moderate methodological quality. One study was categorized as Level I (high quality) and another as Level IV (low quality), respectively. This distribution supports a predominantly moderate- to high-quality evidence base for the meta-analysis

ROBINS-I assessment confirms that the majority of included studies were at low risk of bias, with only two studies showing moderate risk in the domain of participant selection (Fig. 4). Furthermore, the GRADE analysis demonstrates that the majority of included studies were of moderate to high methodological quality (Levels II–III), supporting the credibility of our findings (Fig. 5).

## Results

### Effect of intervention

The primary outcome measured was total radiation time in MIS for orthopedic trauma, guided either by a robotic system or manual fluoroscopy. A comprehensive review of the literature from 2000 onward resulted in the inclusion of ten studies, comprising 675 patients. These studies covered various orthopedic trauma procedures, including fracture alignment and cannulated screw placement. The results revealed a statistically significant reduction in total radiation time in robot-guided procedures compared to manual techniques.

On average, the radiation time per procedure was 203.48 ± 148.34 s for robot-guided surgeries, while manual fluoroscopy guidance resulted in 533.68 ± 176.90 s. A random-effects model was applied to calculate effect size and account for variability across studies. As shown in Fig. 2, the pooled effect size was − 2.01 (95% CI: − 2.74 to − 1.29), exceeding the threshold for a large effect (> 0.828).

This result was supported by a robust fragility index of 240, indicating that 240 additional patients undergoing manual fluoroscopy would need to demonstrate shorter radiation times to reverse the significance. A fragility quotient of 0.38 further reinforced the reliability of this finding, as those 240 patients represent more than one-third of the total sample.

### Heterogeneity analysis

The studies were also examined for heterogeneity using multiple statistical measures. The *τ*^2^ value, representing the overall variation in effect sizes, was significant at 1.241. These findings suggest that the included studies exhibited heterogeneity beyond chance differences. The *I*^2^ value was 92.7%, indicating that 92.7% of the variance between studies could be attributed to heterogeneity rather than random variation. As demonstrated in “Data analysis”, subgroup analysis by type of procedure found a generally low heterogeneity, with Groups 2 and 3 showing *I*^2^ at 0, while Group 1 had a high heterogeneity of *I*^2^ at 96.5%. Overall publication bias, which encompasses bias due to heterogeneity or a disproportionately large effect size due to a small sample, had a 95% CI effect size from − 3 to − 1, indicating no bias.

A random-effects meta-analysis placed the composite effect size in relation to its standard deviation, yielding an H ratio of 3.703. This large value indicates a strong margin of effect in favor of robot-guided surgery.

### Study-level variation and contextual trends

Although robotic guidance consistently reduced fluoroscopy time, the magnitude of reduction varied across procedures and study populations. TiRobot was the most commonly used platform, providing a degree of standardization across studies (see Table 4). However, reductions ranged from 8.1% in thoracolumbar screw placement [9] to 57.4% in intertrochanteric hip fracture repair [27]. These differences likely reflect variation in case complexity, patient age, bone quality, and surgical team experience.

Two studies focused on population-specific contexts, one on younger patients with uncomplicated anatomy [8] and another on older adults with significant bone degeneration [21]. Despite these differences, the studies shared consistent methodologies, including robotic navigation, screw instrumentation, and procedural workflows, enhancing the generalizability of the radiation reduction findings.

This review also builds on prior work, such as the systematic review by Zhang et al. (2022), which reported lower radiation exposure to surgical staff during robot-assisted vertebral augmentation compared to manual techniques, even when patient exposure remained constant [6]. These findings underscore the need to evaluate radiation exposure separately for providers and patients in future studies.

## Discussion

This meta-analysis reinforces the clinical utility of robotic assistance in orthopedic trauma surgery by demonstrating a substantial reduction in total fluoroscopy time. Across ten studies and 675 patients, robot-guided procedures showed a pooled mean radiation time of approximately 203 s, compared to over 530 s with manual guidance. The resulting pooled effect size (Cohen’s *d* = – 2.01, 95% CI: – 2.74 to – 1.29) represents a large and statistically robust reduction, supported by a fragility index of 240 and a fragility quotient of 0.38.

These findings highlight the value of robotic systems in minimizing intraoperative radiation exposure, not only for patients but also for surgical staff. Given the occupational risks associated with cumulative radiation, this reduction has meaningful implications for procedural safety and workflow optimization. Robotic guidance likely improves precision, decreases the need for imaging retries, and reduces interruptions caused by intraoperative adjustments, benefits that align with the institutional goals of efficiency and radiation mitigation.

Heterogeneity analysis revealed variability primarily within procedures involving femoral and acetabular fractures (Group 1, *I*^*2*^ = 96.5%), while vertebral fusion and pelvic fixation subgroups (Groups 2 and 3) showed complete homogeneity (*I*^*2*^ = 0%). These subgroup findings suggest that anatomical complexity and procedural variability drive between-study differences. Nevertheless, the consistently large effect sizes across subgroups and the absence of publication bias (95% CI of funnel plot: – 3 to – 1) reinforce the general reliability of robot-guided fluoroscopy reduction.

Notably, the review builds upon and extends prior work, including findings by Zhang et al., which focused on radiation exposure among surgical staff. The present study adds granularity by evaluating both procedural types and patient contexts, offering a more comprehensive perspective on radiation-saving benefits.

## Limitations

Despite these promising results, several limitations should be considered. First, although fluoroscopy time is commonly reported as an outcome, it serves only as a surrogate for actual radiation dose and does not capture key dosimetric parameters such as absorbed dose, beam intensity, or dose–area product (DAP). Standardization in reporting actual radiation exposure metrics is needed to better understand the clinical impact.

Second, the overall heterogeneity (*I*^*2*^ = 92.7%, *τ*^2^ = 1.24) limits the interpretability of the pooled estimate, although subgroup analysis partially mitigated this by identifying procedure-specific trends. The use of a random-effects model accounts for this variability statistically, but does not eliminate it as a confounding factor.

Third, variation in robotic platforms (despite TiRobot being most common), surgical teams, and institutional protocols may have influenced outcomes. The observed range in radiation reduction, from 8.1% to 57.4%, reflects real-world diversity in implementation and patient characteristics, such as bone quality and fracture complexity. Two studies even targeted highly divergent populations, young adults versus elderly osteoporotic patients, raising questions about the differential efficacy in specific subgroups.

Finally, while the methodological quality of the included studies was generally moderate to high (GRADE Levels II–III) and the ROBINS-I assessment found minimal risk of bias, the small number of studies per subgroup and lack of individual patient-level data limited the ability to perform more nuanced meta-regressions.

## Conclusion

Robotic guidance in orthopedic trauma surgery offers clear advantages in reducing fluoroscopy time, a critical factor in minimizing occupational radiation exposure. While not yet universally adopted as the standard of care, robotic systems consistently demonstrate improvements in procedural efficiency and intraoperative safety across diverse trauma applications.

Despite some heterogeneity across included studies, the magnitude of the pooled effect size and supporting fragility metrics provide robust evidence that robotic assistance significantly reduces radiation exposure during surgery. Future research should aim to disentangle the specific drivers of procedural efficiency, evaluate adoption across varied clinical settings, and optimize protocols that consider cost-effectiveness, training demands, and long-term outcomes.

As robotic platforms and imaging technologies continue to evolve, the goal of minimizing radiation exposure without compromising surgical precision remains central to advancing orthopedic trauma care.

## Data Availability

All data generated or analyzed in this study are included in the published article.
